# Reanalysis of the immunological characteristics of scalp psoriasis: a cross-sectional study using Olink proteomics

**DOI:** 10.3389/fmed.2026.1842465

**Published:** 2026-07-01

**Authors:** Liping Zhu, Wei Chen, Ningning Shen, Qiang Dong

**Affiliations:** Department of Dermatology, Dermatology Hospital of Zhejiang Province, Huzhou, China

**Keywords:** inflammatory, Olink, proteomic, psoriasis vulgaris, scalp

## Abstract

**Objective:**

To analyze the molecular profile of adult patients with Scalp psoriasis (SP).

**Methods:**

We assessed 92 inflammatory biomarkers in lesional scalp skin from SP patients (*n* = 20) and demographically comparable healthy controls (HCs, *n* = 12) using Olink high-throughput proteomics, and investigated the expression of Th2-type cytokines using ELISA in an independent cohort (contains 11 SP patients and 5 HCs).

**Results:**

We identified 28 differentially expressed proteins (DEPs) in lesional SP compared to HCs (FC ≥ ±1.2, adjusted *p*-value <0.05). We observed that SP exhibits a Th1/Th17/IL-12/IL-23-dominant molecular signature. GO clustering and KEGG pathway analyses demonstrated close interrelationships among cytokines, chemokines, and their receptors during the psoriatic inflammatory cascade. Moreover, our results indicated a down-regulation of Th2-type molecules in the SP group, evidenced by significantly reduced levels of IL-4 and IL-5. Notably, these decreases were negatively correlated with the scalp-specific Investigator’s Global Assessment (ss-IGA) scores (IL-4: *r* = −0.78, *p* < 0.01; IL-5: *r* = −0.65, *p* < 0.05), which may lead to an imbalance characterized by an enhanced Th1/Th17/IL-12/IL-23 inflammatory response.

**Conclusion:**

This proteomic profile provides a new perspective on the immunological pathogenesis of SP, suggesting an imbalance between Th2 and Th1/Th17/IL-12/IL-23 responses.

## 1 Introduction

Psoriasis is a common, chronic, immune-mediated skin disease, with a prevalence reported worldwide ranging from 0.12% to 3.2% (1, 2). In addition to involving the trunk and limbs, the disease often affects the scalp (52.8%–80%) (3). Scalp psoriasis (SP) is often accompanied by scaling and pruritus, causing significant psychosocial distress (4). Clinically, SP is sometimes the initial manifestation and the only affected area of psoriasis (5), and is easily confused with seborrheic dermatitis (SD). Although several systemic treatments have been proposed for these “difficult-to-treat” cases (6, 7), efficacy varies among different drugs, and the risk of severe adverse events (AEs) remains a concern (8). These clinical issues prompted us to conduct further research on the molecular mechanisms of SP.

Mechanistically, it is crucial to study the scalp as a unique microenvironment rather than simply an extension of classic plaque psoriasis (psoriasis vulgaris, PV). The scalp is distinguished by its high density of hair follicles and sebaceous glands, which fundamentally alter the local immune landscape. Notably, sebaceous glands not only serve as a physical and antimicrobial barrier in sebaceous gland-rich regions (9), but also secrete lipids that actively modulate local immune responses. Interactions between specific microbes such as Malassezia, sebaceous lipids, and the local immune system can contribute to SP pathogenesis (10, 11) and disease severity (12). Furthermore, hair follicles harbor a rich reservoir of stem cells and resident immune cells, which may drive unique cytokine profiles (13, 14) and contribute to the recalcitrance of SP to conventional therapies (15, 16). Therefore, elucidating the distinct molecular drivers of SP is essential for understanding its unique pathophysiology and developing targeted therapeutic strategies.

Few studies have explored the immunological characteristics of SP. Two publications focused on comparing scalp psoriasis with psoriasis vulgaris (PV) using transcriptomic analyses and identified differences and similarities between them (17, 18). However, one study conversely suggested that PV shares similar cellular, molecular, and barrier characteristics with SP at both gene and protein levels (19). These results indicate that the pathophysiology of scalp psoriasis is not fully understood and may possess unique features, necessitating further exploration.

Recently, Olink proteomics have been used to investigate skin diseases, primarily focusing on atopic dermatitis (AD) (20, 21), scalp seborrheic dermatitis (SSD) (22), alopecia areata (AA) and psoriasis (23), and hidradenitis suppurativa (HS) (24, 25). Most of these studies used blood samples rather than skin samples. Consequently, data regarding Olink platforms applied to scalp psoriasis are lacking. Therefore, we aimed to investigate the proteomic features of SP in lesional skin using the Olink platform. Our data will provide a new perspective on the molecular characteristics.

## 2 Materials and methods

### 2.1 Patient enrollment

This study was approved by the Institutional Review Board of Dermatology Hospital of Zhejiang Province (Approval No. 2025-02K). All methods were performed in accordance with relevant guidelines and regulations. Written informed consent was obtained from all participants. Untreated adult patients with scalp psoriasis (aged ≥18 years) and demographically comparable healthy controls (HCs) were enrolled for Olink and ELISA (see Tables 1, 2). Scalp psoriasis was diagnosed through clinical evaluation by two senior dermatologists, supplemented by dermoscope examination. Disease severity was assessed using the scalp-specific Investigator’s Global Assessment (ss-IGA), a 5-point scale grading from 0 (no disease) to 4 (severe disease). The inclusion criteria required that patients had not received systemic immunosuppressants, biological agents, or phototherapy within the preceding 3 months, and had not used topical medications within the preceding 1 month. Patients with other inflammatory skin diseases, such as seborrheic dermatitis and atopic dermatitis (AD), were excluded.

**Table 1:** Patient demographics of scalp psoriasis and HCs in Olink research.

Characteristics	Scalp psoriasis (*n* = 20)	Healthy controls (*n* = 12)	*p*-value
Age (y)			
Mean (SD)	50.1 (13.6)	40.8 (14.4)	0.08
Range	25–69	24–68	
Sex, *n* (%)			
Male	13 (65.0)	7 (58.3)	0.72
Female	7(35.0)	5 (41.7)	
BMI			
Mean (SD)	24.7 (3.2)	23.0 (3.1)	0.14
Range	20.0–31.3	19.6–30.5	
Sleep time			
Mean (SD)	6.7 (2.2)	7.5 (0.9)	0.16
Range	3–12	6–9	
Sleep quality			
Normal	11 (55.0)	9 (75.0)	0.45
Poor	9 (45.0)	3 (25.0)	
Course (*y*)			
Mean (SD)	10.6 (9.2)		
Range	1–30		
ss-IGA score			
Mean (SD)	2.8 (0.8)		
Range	2–4		

Quantitative comparisons were evaluated using the un-paired students’ *t*-tests. Analogous comparisons were assessed with Fisher’s exact tests. BMI, body mass index.

**Table 2:** Patient demographics of Scalp Psoriasis and HCs in ELISA.

Characteristics	Scalp psoriasis (*n* = 11)	Healthy controls (*n* = 5)	*p*-value
Age (y)			
Mean (SD)	53.0 (12.2)	62.8 (7.0)	0.12
Range	37–73	56–74	
Sex, *n* (%)			
Male	7 (63.6)	3 (60.0)	0.65
Female	4 (36.4)	2 (40.0)	
BMI			
Mean (SD)	26.1 (2.1)	24.8 (2.9)	0.32
Range	23.2–29.6	20.8–28.7	
Sleep time			
Mean (SD)	6.9 (1.4)	6.6 (0.5)	0.64
Range	5–9	6–7	
Sleep quality			
Normal	8 (72.7)	4 (80.0)	0.64
Poor	3 (27.3)	1 (20.0)	
Course (*y*)			
Mean (SD)	7.3(7.2)		
Range	0.3–20		
ss-IGA score			
Mean (SD)	3.3(0.8)		
Range	2–4		

Quantitative comparisons were evaluated using the un-paired students’ *t*-tests. Analogous comparisons were assessed with Fisher’s exact tests. BMI, body mass index.

### 2.2 Skin sample collection

Participants were sampled at baseline. Lesional punch biopsies (3 mm) were obtained from active inflammatory sites on the scalps of patients for Olink and ELISA. The skin tissues were immediately placed in 5-mL Eppendorf tubes, snap-frozen in liquid nitrogen, and stored at −80 °C until analysis.

### 2.3 Skin protein quantification

Skin tissues were lysed in RIPA lysis buffer [50 mM Tris (pH 7.4), 150 mM NaCl, 1% NP-40, 0.25% sodium deoxycholate, sodium orthovanadate, sodium fluoride, EDTA, leupeptin] supplemented with protease inhibitors at a 1:1000 ratio. Stainless-steel beads (0.21 g) were added to each sample, which was then homogenized using a tissue grinder at 2 °C and 60 Hz. The grinding protocol consisted of 60 cycles of 10 s each, with a 10-s interval between cycles. The homogenates were subjected to ultrasonic extraction using a non-contact ultrasonic homogenizer. After centrifugation at high speed (12,000 × *g*) and low temperature (4 °C) for 15 min, the supernatants were collected. Protein concentration was determined using the BCA method. Finally, samples were diluted to 1 μg/μL for Olink analysis using the Inflammation Panel, as previously described (20, 21, 25, 26).

### 2.4 Data processing protocol and QC system

Protein measurements were generated using the Olink Target Inflammation panel (v.3027) and processed with Olink NPX Signature (v1.16.0). The NPX values represent normalized relative protein abundance on a log_2_ scale. Values below the lower limit of detection (LOD) were treated as null. Four internal controls are designed to evaluate and monitor the distinct stages of the PEA process, including two incubation/immuno controls, an extension control, and a detection control. These internal controls are added to both study samples and external controls to ensure quality assurance and data normalization. External quality control involves eight samples (two positive controls, three negative controls, and three inter-plate controls). Specifically, the negative controls are used for LOD determination, while the positive and inter-plate controls are utilized for calculating intra- and inter-batch CVs, as well as for data normalization.

### 2.5 Bioinformatic analysis

Gene ontology (GO) functional annotation and Kyoto Encyclopedia of Genes and Genomes (KEGG) pathway enrichment analyses were performed on the selected differentially expressed proteins (DEPs) using R software (version 4.0) (27, 28).

### 2.6 Statistical analysis

Differential expression analysis of the Olink proteomic data was performed using the “Limma” package in R software (version 4.0.1). We applied the Benjamini–Hochberg method using R’s limma framework to control the False Discovery Rate (FDR). The results reported in the manuscript include both raw *p*-values and adjusted *p*-values (FDR) to ensure rigor and avoid type I errors. The fold change (FC) ≥ ±1.2 and adjusted *p*-value < 0.05 were used as the thresholds for selection of DEPs. For quantitative comparisons between two groups, a two-sided, unpaired Student’s t-test was performed. Analogous comparisons were assessed chi-square test, with Fisher’s exact tests. Statistical significance was defined as *p* < 0.05. Pearson correlation coefficients were used to calculate the correlations between differential expressed proteins and clinical severity (measured by ss-IGA).

## 3 Results

### 3.1 Participant characteristics and proteomic profiling

We enrolled 20 adult patients with SP and 12 HCs. No significant differences were observed in age, sex, body mass index (BMI), sleep duration, or sleep quality between the SP and HC groups (Table 1). A total of 60 inflammatory proteins were detected in scalp skin tissues, with no significant difference in the total number of detected proteins between the groups (Supplementary Figure S1). Principal component analysis (PCA) demonstrated that lesional skin samples from SP patients clustered separately from those of HCs (Supplementary Figure S2).

### 3.2 The proteomic profile of scalp psoriasis exhibits a Th1/Th17/IL-12/IL-23-dominant molecular signature

**Figure 1:**
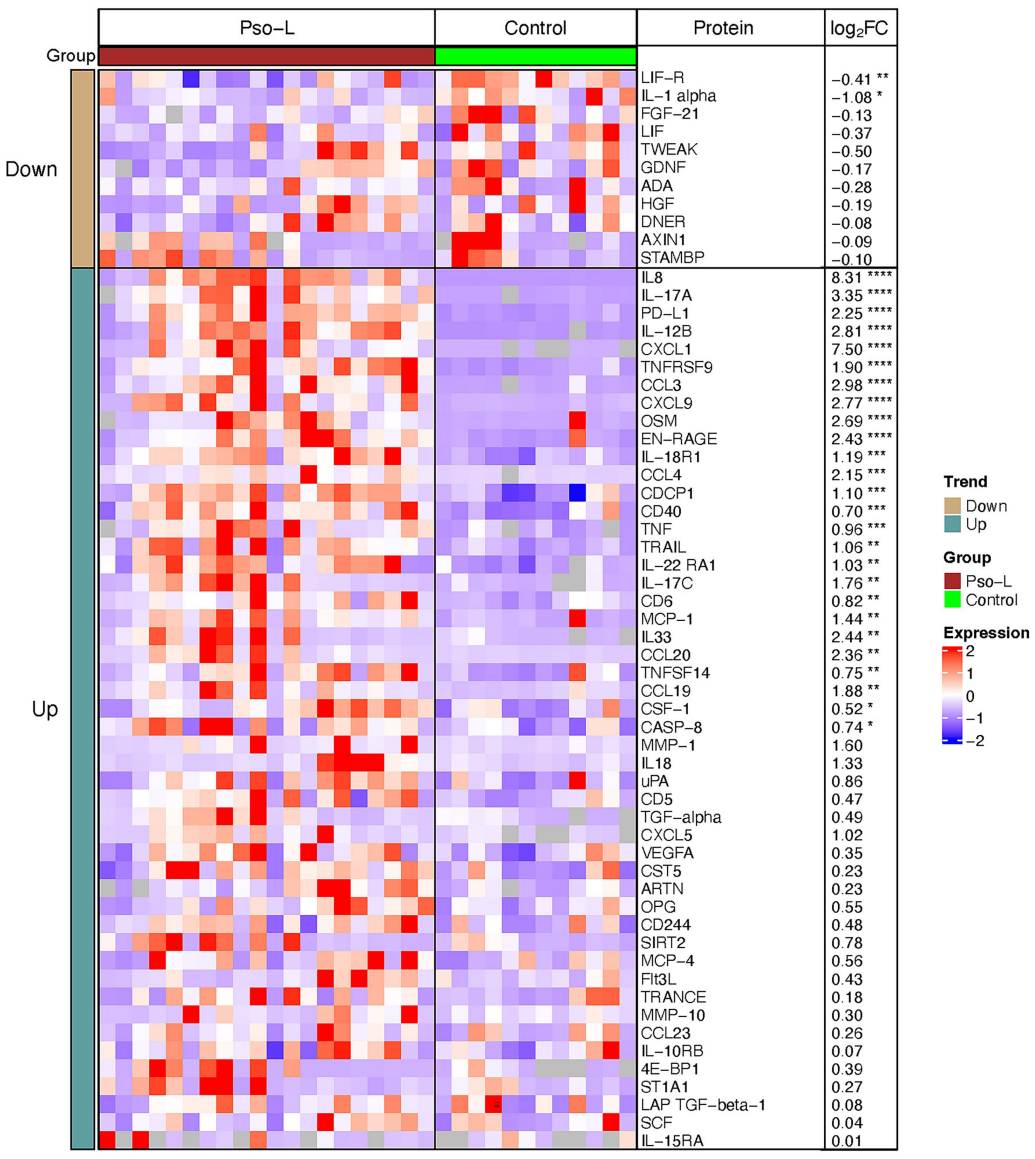
Heatmap of 28 DEPs (FC ≥ ±1.2, adjusted *p*-value <0.05) in the lesional skin of SP compared to healthy controls (HCs). Each column represents an individual patient. Lesional scalp (L), healthy controls (C).

We identified 28 differentially expressed proteins (DEPs) in lesional scalp skin compared to HCs (FC ≥ ±1.2, adjusted *p*-value <0.05) (Figure 1). Among these, IL-1α and LIF-R were down-regulated, while the remaining 26 proteins were up-regulated. SP lesions exhibited significant upregulation of proteins associated with Th1 (IL-8, CCL3, CCL4, CCL19, CCL20, CXCL9, IL-18R1, TNFSF14, CASP-8, CD40, CSF-1, CD6), IL-12/IL-23 (CCL3, CXCL9, TNF, IL-17A, IL-17C, IL-12B, CD40, CCL19), Th22 (IL-22RA1), Th17 (CXCL1, IL-17A, IL-17C, CCL20), neutrophil chemotaxis (CXCL1, IL-8), Th2 (IL-33) (Supplementary Table S1). IL-8 and CXCL1 showed the highest fold changes, reaching 317 and 181, respectively. Notably, most Th2-related cytokines, such as IL-4, IL-5, IL-10, IL-10RA, and IL-13, were rarely detected in SP lesional skin using the Olink platform (Below the detection threshold), therefore, these molecules were not included in the functional analysis. Further Pearson correlation analysis results did not find a relationship between these differential expressed proteins and clinical severity (Supplementary Table S2).

### 3.3 Functional analysis of DEPs

**Figure 2:**
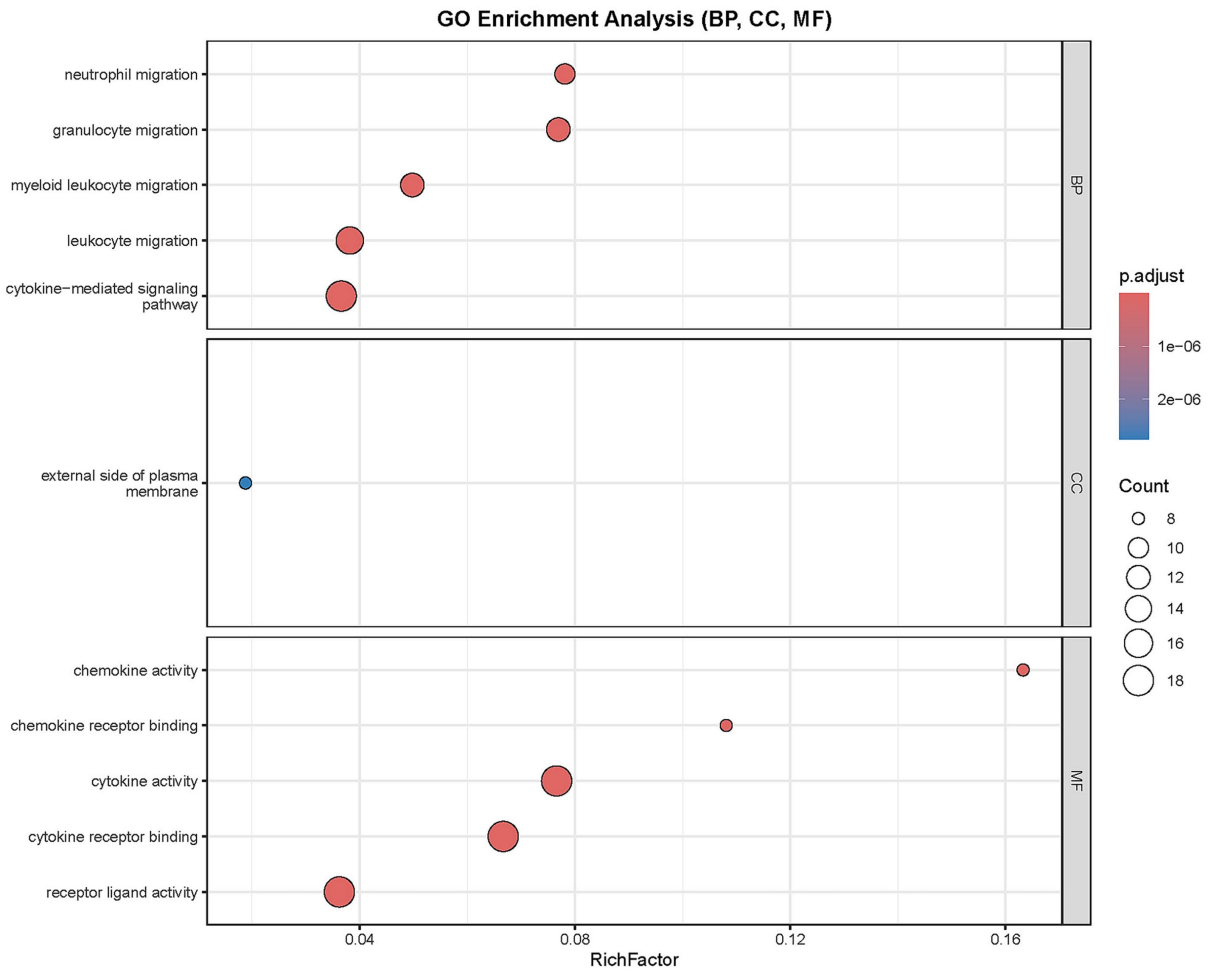
GO enrichment analyses were performed in 28 DEPs (FC ≥ ±1.2, adjusted *p*-value <0.05). Dotplot displayed the top 5 significant difference GO terms (The Cellular Component (CC) enriches only one cellular location). An adjusted *p*-value < 0.05 was used for terms selection. The color of the dots represents the adjusted *p*- value (p.adjust), with redder colors indicating lower *p*- values (higher statistical significance). The size of the dots corresponds to the count of proteins enriched in each GO term, where larger dots signify a greater number of enriched proteins.

**Figure 3:**
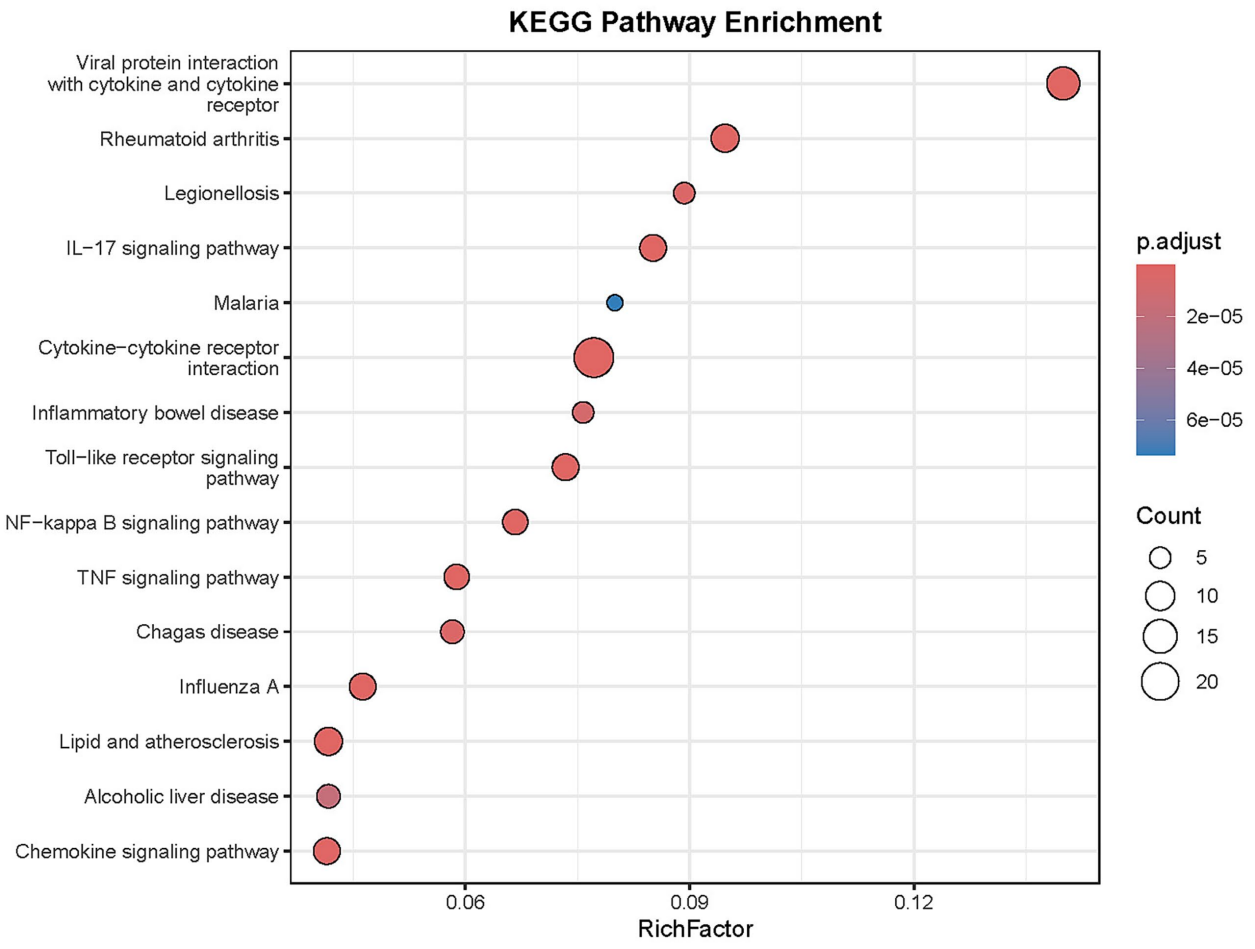
KEGG enrichment analyses were performed in 28 DEPs (FC ≥ ± 1.2, *p*-value < 0.05). Dotplot displayed the top 15 enriched pathways in Kyoto Encyclopedia of Genes and Genomes (KEGG) enrichment analysis (b). An adjusted *p*-value < 0.05 was used for pathway selection. Bubble size indicates gene count, and bubble color reflects adjusted *p*-values, ranging from red (lower *p*-value) to blue (higher *p*-value).

To elucidate the biological functions of the DEPs, we performed Gene Ontology (GO) enrichment and KEGG pathway analyses. GO analysis revealed significant enrichment in chemokine activity, chemokine receptor binding, cytokine activity, and cytokine receptor binding (Figure 2). KEGG pathway analysis indicated that these proteins are primarily involved in psoriasis-related signaling pathways, including the IL-17 signaling pathway, TNF signaling pathway, chemokine signaling pathway, and cytokine-cytokine receptor interaction (Figure 3). These results demonstrate a close interplay among cytokines, chemokines, and their receptors during the psoriatic inflammatory cascade.

### 3.4 Validation of Th2-type cytokine expression in SP using ELISA

Given that Th2-related cytokines (IL-4, IL-5, IL-10, and IL-13) were rarely detected in the Olink assay, we further investigated their expression levels using ELISA in an independent cohort consisting of 11 SP patients and 5 HCs. No significant differences were observed in age, sex, BMI, sleep duration, or sleep quality between the SP and HC groups (Table 2). There were no significant statistical differences in the primary baseline characteristics of SP patients in this cohort compared with those enrolled in the Olink study in terms of age, disease duration, BMI and ss-IGA score (*p* = 0.56, 0.13, 0.20 and 0.32 respectively). ELISA results indicated that the levels of IL-4 and IL-5 were significantly decreased in the SP group compared to HCs (*p* = 0.03 and *p* = 0.02, respectively), whereas IL-10 and IL-13 levels showed no significant change (*p* = 0.24 and *p* = 0.42, respectively) (Supplementary Table S3).

We then performed Pearson correlation analyses to explore the associations between Th2-related cytokines (IL-4, IL-5, IL-10, and IL-13) and ss-IGA score. IL-4 (*r* = −0.78, *p* < 0.01), IL-5 (*r* = −0.65, *p* < 0.05) and IL-13 (*r* = −0.66, *p* < 0.05) have a negative correlation with the ss-IGA, while IL-10 has no significant correlation with ss-IGA (Supplementary Table S4). These finding suggest that Th2-type molecular activity is suppressed or absent, potentially contributing to an imbalance characterized by an enhanced Th17/IL-12/IL-23 inflammatory response.

## 4 Discussion

In this study, we examined the molecular profile of SP through a minimally invasive 3 mm trephine using Olink high-throughput proteomics and investigated the expression of Th2-type cytokines using ELISA in an independent cohort. We observed that SP exhibits a Th1/Th17/IL-12/IL-23-dominant molecular signature. GO clustering and KEGG pathway analyses demonstrated close interrelationships among cytokines, chemokines, and their receptors during the psoriatic inflammatory cascade. Moreover, our results indicated a down-regulation of Th2-type molecules in the SP group, evidenced by significantly reduced levels of IL-4 and IL-5. Notably, these decreases were negatively correlated with the scalp-specific Investigator’s Global Assessment (ss-IGA) scores (IL-4: r = −0.78, *p* < 0.01; IL-5: *r* = −0.65, *p* < 0.05), which may lead to an imbalance characterized by an enhanced Th1/Th17/IL-12/IL-23 inflammatory response.

To date, only a few studies have compared the immune features of psoriasis across different skin areas (17–19, 25, 29–31), with only three publications specifically focusing on comparing SP with PV. Ahn et al. (17) found that distinct psoriasis types exhibited differences in IL-17, IFN-*γ*, and IL-22 expression; however, these differences were primarily significant when compared to palmoplantar psoriasis. Ruano et al. (18) revealed shared immune mechanisms between SP and PV through transcriptomic analyses, but the quantity of differentially expressed genes (DEGs) and psoriatic genomic fingerprint enrichment were more prominent in PV. Additionally, Gáspár et al. (19) showed that mediators of both innate immune responses and Th1/Th17-type adaptive immunity were expressed similarly in SP and PV using immunohistochemistry and RT-qPCR. In general, our results align with these studies, confirming that psoriatic lesional skin demonstrates significant upregulation of Th1/Th17/IL-12/IL-23 proteins, consistent with findings in psoriasis vulgaris (24), despite the diversity of methods used.

Studies have established that Th2-type molecules do not play a dominant role in psoriasis, clearly distinguishing it from atopic dermatitis (AD) (32). Tsoi et al. (33) identified distinct gene signatures separating the two diseases, notably IL-13/IL-4 responses in AD versus IL-17 responses in psoriasis. Consistent with these findings, our proteomic analysis detected minimal Th2-type molecule activity. Further ELISA results indicated that Th2 molecule expression was either downregulated (IL-4, IL-5) or showed no significant change (IL-10, IL-13), aligning with a transcriptome study conducted using tape strips from lesional and non-lesional skin (34).

Notably, IL-33 was highly expressed in this study. While IL-33 can be induced by various cytokines—including IFN-*γ* (Th1), IL-17 (Th17), IL-4, and IL-13 (Th2)—in human keratinocytes, it acts as a chemoattractant for Th2 cells and an ‘alarmin’ that amplifies immune responses. Given the observed downregulation or absence of most Th2 molecules, we speculate that the high expression of IL-33 is primarily driven by Th1 and Th17 activation (35). Furthermore, IL-33 may contribute to the pathogenesis of psoriasis by inducing CCL20 production, which recruits Th17 cells and CXCL8 to the epidermis (35), rather than eliciting a Th2 inflammatory response. Although a study in a Chinese population indicated a strong Th2 component (e.g., IL-4, IL-13, IL-25, IL-31, and TSLP) in psoriatic lesions, the proportion of such samples in the cluster was relatively low (6/40) (36). Our research revealed that IL-4 and IL-5 were significantly decreased in the SP group and have a negative correlation with the ss-IGA. These results collectively suggest that Th2-type activity is suppressed in SP, contributing to an imbalance that favors an enhanced Th17/IL-12/IL-23 inflammatory response. Supporting our findings, methotrexate treatment has been shown to upregulate Th2 components and induce a shift from a Th1 to a Th2 pathway in plaque-type psoriasis (37, 38). Conversely, IL-4/IL-13 inhibition (Dupilumab) for AD can induce psoriatic rash, driven by a shift from Th2 to IL-36 and Th17 polarization (39). In conclusion, the role of Th2-type molecules in scalp psoriasis requires further study with large sample data.

Our data identified IL-8 and CXCL1 as the molecules with the highest fold-change upregulation in SP lesional skin. CXCL1, a neutrophil chemokine implicated in psoriasis (40), has been identified as a hub gene in mild psoriasis (41). Its elevated expression in keratinocytes may be triggered by IL-17, TNF-*α*, and IL-36*γ*, leading to neutrophil infiltration into psoriatic lesions (42), where they produce IL-17 and secrete reactive compounds. Similarly, IL-8 (CXCL8) attracts neutrophils and T-cells (43, 44). Since CXCL1 and IL-8 share the receptor CXCR2 (45), both chemokines likely contribute to neutrophil recruitment and infiltration in PV (46). Previous research indicates that CXCL1 and IL-8 levels normalize following treatment with Bimekizumab (an antibody inhibiting IL-17A and IL-17F) (47) and Spesolimab (an anti-IL-36 receptor antibody) (48).

### 4.1 Limitations of the study

(1) Our analysis was limited to 92 proteins. (2) Potential for disease misclassification, such as seborrheic dermatitis. (3) This cross-sectional study only characterized SP in the adult population. (4) Furthermore, the relatively small sample size (*n* = 20 for SP) may limit the statistical power to detect subtle proteomic changes and increase the risk of type II errors. (5) The Th2 molecules that failed to be detected in Olink detection (Below the detection threshold) may be limited by the sample processing procedure.

## 5 Conclusion

In this study, we examined the molecular profile of SP using Olink high-throughput proteomics and investigated the expression of Th2-type cytokines using ELISA in an independent cohort. We observed that SP exhibits a Th1/Th17/IL-12/IL-23-dominant molecular signature. GO clustering and KEGG pathway analyses demonstrated close interrelationships among cytokines, chemokines, and their receptors during the psoriatic inflammatory cascade. Moreover, our results indicated a down-regulation of Th2-type molecules in the SP group, evidenced by significantly reduced levels of IL-4 and IL-5. Notably, these decreases were negatively correlated with the scalp-specific Investigator’s Global Assessment (ss-IGA) scores, which may lead to an imbalance characterized by an enhanced Th1/Th17/IL-12/IL-23 inflammatory response. This proteomic profile provides a new perspective on the immunological pathogenesis of SP.

## Data Availability

The datasets presented in this study can be found in online repositories. The names of the repository/repositories and accession number(s) can be found in the article/Supplementary material.
