# Supplementary figures and images for: Reanalysis of the immunological characteristics of scalp psoriasis: a cross-sectional study using Olink proteomics
Source: Front Med (Lausanne). 2026 Jul 1;13:1842465. doi: 10.3389/fmed.2026.1842465 (PMC13368496; doi:10.3389/fmed.2026.1842465)

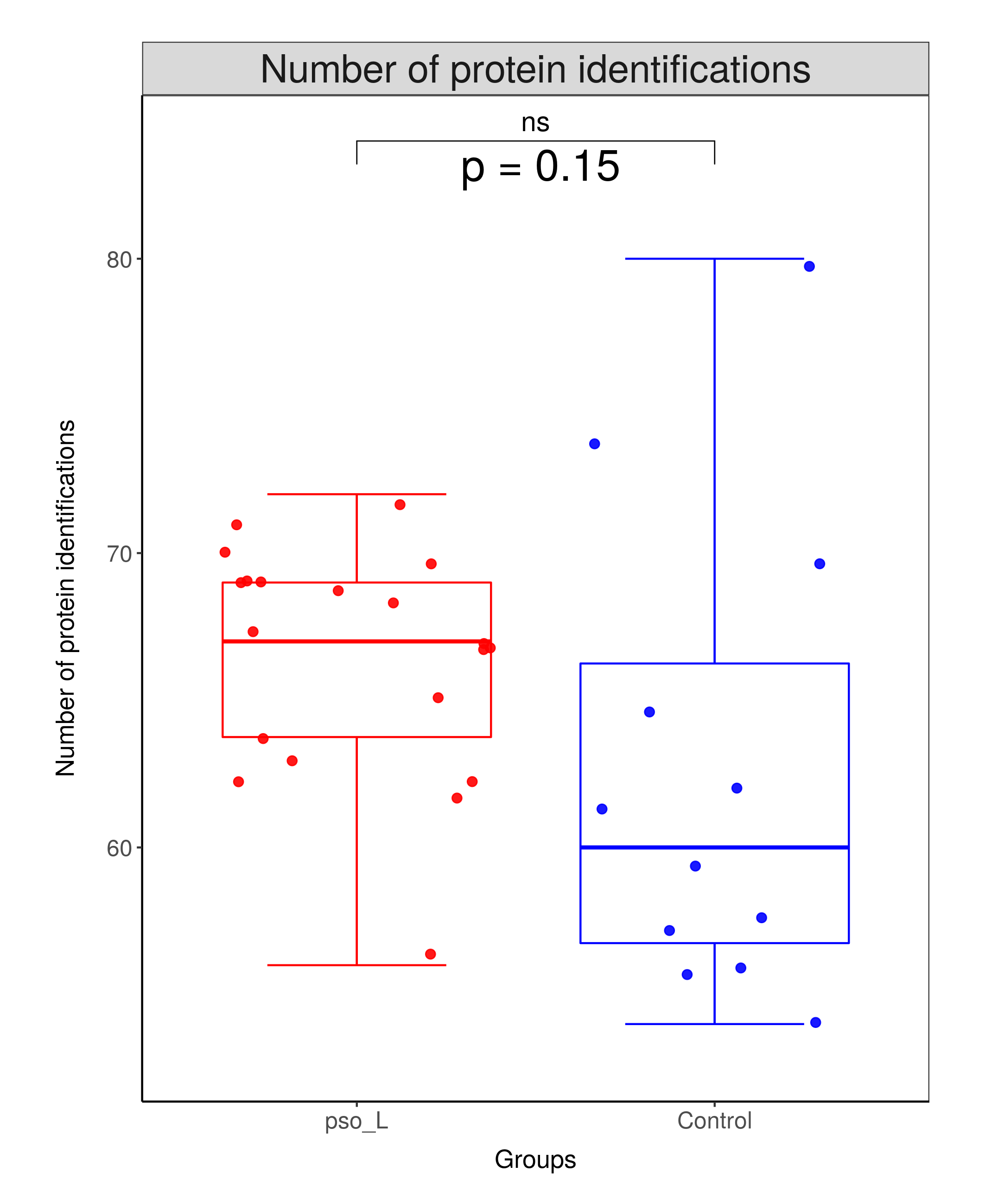

Supplement: Supplementary file 1 [file Image_1.PNG]

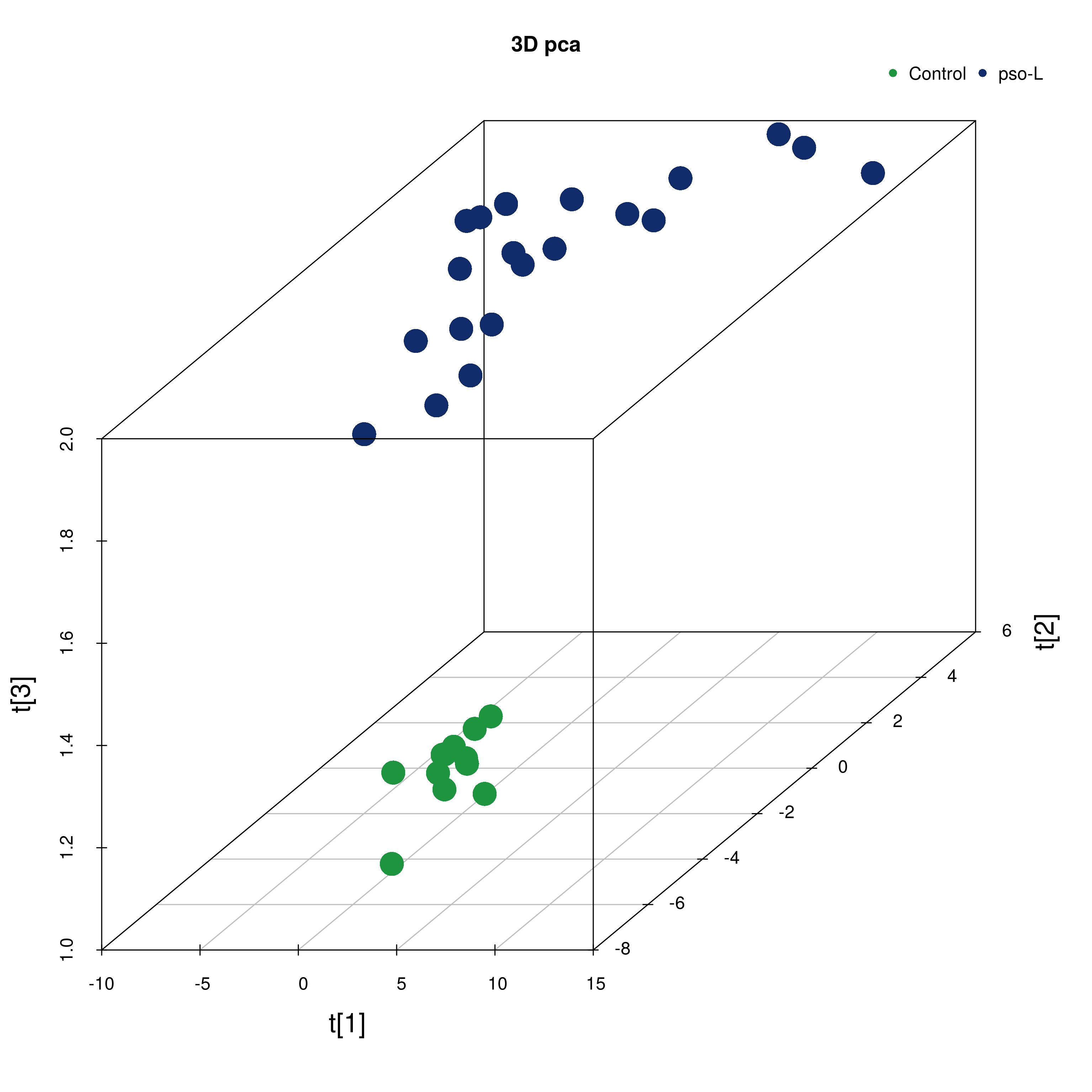

Supplement: Supplementary file 2 [file Image_2.PNG]
